# Vaccinia Protein F12 Has Structural Similarity to Kinesin Light Chain and Contains a Motor Binding Motif Required for Virion Export

**DOI:** 10.1371/journal.ppat.1000785

**Published:** 2010-02-26

**Authors:** Gareth W. Morgan, Michael Hollinshead, Brian J. Ferguson, Brendan J. Murphy, David C. J. Carpentier, Geoffrey L. Smith

**Affiliations:** Department of Virology, Faculty of Medicine, Imperial College London, London, United Kingdom; Saint Louis University, United States of America

## Abstract

Vaccinia virus (VACV) uses microtubules for export of virions to the cell surface and this process requires the viral protein F12. Here we show that F12 has structural similarity to kinesin light chain (KLC), a subunit of the kinesin-1 motor that binds cargo. F12 and KLC share similar size, pI, hydropathy and cargo-binding tetratricopeptide repeats (TPRs). Moreover, molecular modeling of F12 TPRs upon the crystal structure of KLC2 TPRs showed a striking conservation of structure. We also identified multiple TPRs in VACV proteins E2 and A36. Data presented demonstrate that F12 is critical for recruitment of kinesin-1 to virions and that a conserved tryptophan and aspartic acid (WD) motif, which is conserved in the kinesin-1-binding sequence (KBS) of the neuronal protein calsyntenin/alcadein and several other cellular kinesin-1 binding proteins, is essential for kinesin-1 recruitment and virion transport. In contrast, mutation of WD motifs in protein A36 revealed they were not required for kinesin-1 recruitment or IEV transport. This report of a viral KLC-like protein containing a KBS that is conserved in several cellular proteins advances our understanding of how VACV recruits the kinesin motor to virions, and exemplifies how viruses use molecular mimicry of cellular components to their advantage.

## Author Summary

Vaccinia virus (VACV), the vaccine used to eradicate smallpox, exploits the host cell motor kinesin-1 to export virus particles to the cell surface. We demonstrate that the VACV F12 protein has structural similarity with kinesin light chain (KLC) and facilitates viral transport using a kinesin binding sequence (KBS) that is conserved in several neuronal proteins. Dysfunction of some of these neuronal proteins can contribute to diseases, such as Alzheimer's. Mutation of the KBS in protein F12 showed it is essential for kinesin recruitment to virions and for virion transport to the cell surface. These findings enhance our understanding of how viruses hijack the host cell transport system, demonstrate conservation of a kinesin binding motif in cellular and viral proteins and identify targets for drug development.

## Introduction

Vaccinia virus (VACV) is a large DNA virus that replicates in the cytoplasm [1] and produces morphologically distinct virions that have different roles in virus dissemination [2],[3]. Virus replication occurs in cytoplasmic factories and starts with the formation of membrane crescents, composed of lipid and viral protein. These extend into spherical or oval immature virions (IVs) containing the viral genome and core proteins [4],[5]. IVs undergo proteolytic processing of some capsid proteins to form intracellular mature virus (IMV), which mostly remain intracellular and are released only following cell lysis. However, some IMV are transported on microtubules [6],[7] to a site of membrane wrapping where they obtain a double membrane [8]–[10] derived from early endosomes and/or the *trans*-Golgi network, to form the intracellular enveloped virus (IEV) [11]–[13].

For export of virions to the cell periphery, IEV particles are transported on microtubules utilising kinesin-1 [14]–[17] and this process requires the VACV protein F12 [18]–[20] and is influenced by protein A36 [15],[17],[19], which can bind to KLC *in vitro* [21]. Metazoan kinesin-1 transports cargo toward the plus-end of microtubules and is a heterotetramer comprising two kinesin heavy chains (KHCs) and two kinesin light chains (KLCs). KHC contains an N-terminal motor domain, a long coiled-coil stalk interrupted by a central hinge, and a globular tail domain [22]. The KLCs contain N-terminal heptad repeats, which interact with the central coiled stalk of the KHCs, followed by tetratricopeptide repeats (TPRs) that bind cargo [23]. Both KHCs and KLCs have been implicated in attaching kinesin-1 to cargo [24]. KLCs bind diverse cargos through a 34-amino-acid TPR [25],[26] comprising a repeating pattern of small and large amino acids [27]. Recently, the structure of the KLC2 TPRs was solved by crystallography (pdb ID∶3CEQ). Cargo proteins binding to the KLC-TPR motif include the MAP kinase scaffolding proteins called JIPs (Jun-N-terminal kinase interacting proteins) [28], Kidins220/ARMS [29], calsyntenin/alcadein [30], collapsin response mediator protein-2 (CRMP-2 [31]), Huntington-associated protein-1 [32], torsinA [33], 14-3-3 [34], the amyloid precursor protein of axonal vesicles [35], Cayman ataxia protein caytaxin [36], VACV A36 [17],[21] and the *Salmonella* protein PipB2 [37]. The C-terminal region of KLC also influences cargo binding [38]. Mechanisms for cargo binding to, and release from, the KLC TPR are important aspects of cell biology [39]–[41].

When an IEV particle reaches the cell periphery, it dissociates from kinesin-1 and this process is influenced by phosphorylation of A36 [42] and by VACV protein A33 which binds to the same region of A36 that binds kinesin-1 [21]. IEV then traverses the actin cortex [43] and fuses its outer membrane with the plasma membrane to expose a virus on the cell surface called cell associated enveloped virus (CEV) [2]. A36 is then phosphorylated by tyrosine kinases [44],[45] and this causes recruitment of cell proteins Nck, Grb2, N-WASP and Arp2/3 and polymerisation of actin beneath the CEV particle [42], [44], [46]–[49]. If CEVs are released from the cell they are called extracellular enveloped viruses (EEVs). CEVs are important for cell-to-cell spread, whereas EEVs mediate long-range dissemination of virus. Defects in either microtubule-based transport of IEV or actin tail formation beneath CEV, cause a small plaque phenotype and severe attenuation [2],[3].

This paper concerns the transport of IEV to the cell surface and the roles of F12 and A36 proteins in that process. F12 and A36 are each important for virus transport within or between cells and have been called transport proteins [2]. However, these proteins have different roles. A36 is a membrane protein that is expressed early and late during infection and is important for plaque size and virulence [50]. It has type Ib membrane topology, is localised to the IEV outer membrane [18] and is required for actin tail formation [44],[51],[52]. A36 can bind KLC *in vitro* via aa residues 81–111 [15]. However, A36 is not needed for IEV transport or CEV formation. A deletion mutant lacking gene *A36R*, vΔA36R, produced numerous CEV visible by electron microscopy [14],[51],[52] and confocal microscopy [18],[19]. Furthermore, live video microscopy of EGFP-tagged virions lacking A36 showed that A36 negative IEV are transported to the cell surface at 60% of wild type levels by 24 h p.i. [19]. These IEV moved at speeds characteristic of microtubule-based transport and this movement was inhibited reversibly by microtubule de-polymerising drugs [19]. These observations indicate that VACV must have at least one other protein to bind kinesin-1 for microtubule-based transport. In contrast to these reports, another study concluded A36 was essential for IEV transport [17].

F12 is a 65-kDa protein that lacks transmembrane sequences and is conserved in chordopoxviruses [20]. Disruption of its orthologue in fowlpox virus by insertional mutagenesis caused a small plaque phenotype and a reduction in EEV [53]. A VACV deletion mutant lacking *F12L*, vΔF12L, also produced a small plaque, did not form actin tails and was highly attenuated *in vivo* [20]. Subsequent analysis showed that F12 was associated with IEV particles and was needed for IEV transport but not IEV formation [18],[19]. Recently, F12 was reported to form a complex with VACV protein E2, without which CEV and EEV levels were reduced by 2 logs and the virus formed a small plaque [54],[55]. F12 also interacts with A36 and this interaction was proposed to recruit F12 to IEV [56]. Collectively, these observations show that IEV transport to the cell surface has a greater dependency on F12 than A36.

Here we show that F12 has structural similarity to KLC and contains TPRs and a WD motif that is required for kinesin-1 recruitment and virion transport. In contrast, WD motifs in A36 were not required for either process. Furthermore, TPRs were identified in proteins A36 and E2. We propose that F12 functions as a bridge to bind IEVs to the kinesin-1 motor. F12 interaction with IEV may be mediated by TPR∶TPR contacts between F12, E2/A36, and kinesin-1 binding is mediated by the WD motif found in F12 and cellular kinesin-1 binding proteins.

## Results

### F12 exhibits sequence similarity to KLC cargo-binding tetratricopeptide repeats

**Figure 1:**
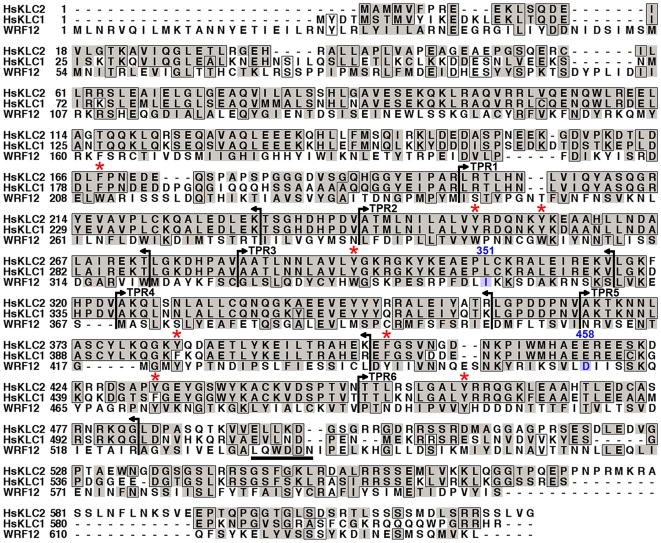
F12 has similarity to KLC. VACV strain WR F12 (WRF12) was aligned with human KLCs 1 and 2 (HsKCL1, HsKCL2). Identical residues are boxed and conserved residues are shaded. Vertical bars and arrows indicate the positions of HsKLC TPRs. The red asterisk above the alignment denotes the conserved aromatic residues in TPRs. The F12 WD motif at aa residues 534 to 538 is underlined and residues 351–458 (blue) have been shown to bind A36 [56]. Accession numbers are WR F12 (AAO89330), HsKLC1 (AA01263), and HsKLC2 (Q9H0B6).

The F12 protein from VACV strain WR F12 (WRF12) is conserved in other VACV strains, orthopoxviruses and chordopoxviruses [20] implying an important function. Previous BLAST analysis failed to reveal sequence similarity between F12 and host proteins [20],[55],[56], but further analysis here identified a pattern of conserved aromatic residues (phenylalanine, tyrosine and tryptophan – Figure 1 red asterisks) and small hydrophobic residues (leucine, proline and valine) similar to the cargo-binding TPRs of KLC (Figure 1, between vertical bars). Indeed, the entire F12 protein could be aligned with human KLC1 and KLC2 (Figure 1). HsKLC1 and HsKLC2 share 61% aa identity (71% aa similarity), but have only 12% aa identity (50% aa similarity) with VACV F12. Nonetheless, these proteins have similar size and charge (HsKLC1, pI 5.94, Mr 64.7 kDa; HsKLC2, pI 6.72 Mr 68.9 kDa; WR F12, pI 5.76, Mr 73.2 kDa), and similar oscillating hydropathy profiles without transmembrane regions (Figure S1). Like KLC, F12 is a cytosolic protein as shown by Triton X-114 partitioning (Figure S2) in agreement with a recent report [56], however, F12 lacks the heptad repeats near the N terminus of KLC for binding KHC.

The TPR (NCBI CDD cd00189) is a common structural motif facilitating protein-protein interactions and the assembly of multi-protein complexes. The repeat comprises a pattern of small and large amino acids [WLF]-X(2)-[LIM]-[GAS]-X(2)-[YLF]-X(8)-[ASE]-X(3)-[FYL]-X(2)-[ASL]-X(4)-[PKE] with no position invariant [27]. Structurally, TPRs form paired helices of two types: an A/B interaction within each helical pair is mediated by conserved small amino acids that zipper the first two helices together; and a B/A′ interaction that links one helix pair to the next, mediated by conserved aromatic residues. TPRs are evolutionarily conserved and a cluster of three consecutive TPRs is the most common organization [27]. Alignment of VACV F12 and related proteins from other chordopoxviruses ([20] and Figure S3) shows a pattern of bulky and intervening small hydrophobic residues that resemble TPRs and are conserved between these proteins. F12 has 14 TPR-like sequences constituting 80% of the protein (Figure S3).

**Figure 2:**
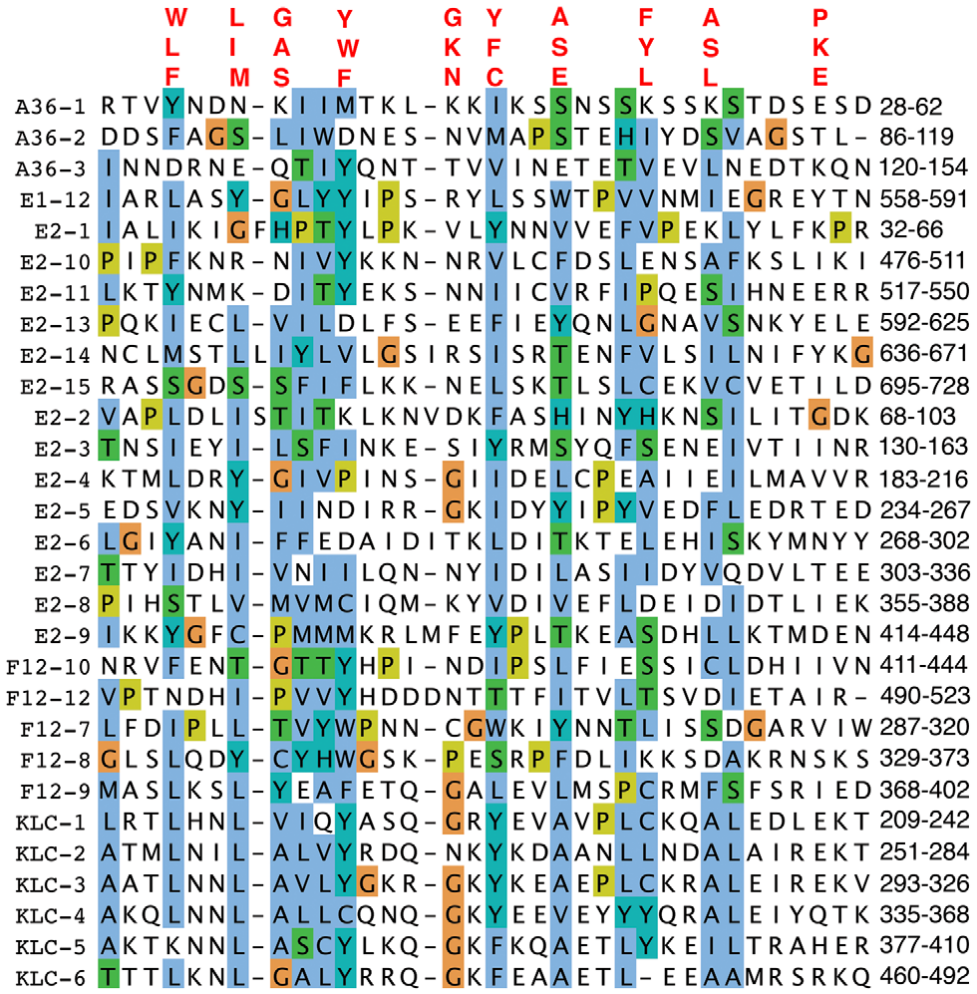
Alignment of TPRs of VACV proteins F12, A36, and E2 with KLC TPRs. The TPRs of A36, E2 and F12 were aligned using Clustal-X and are displayed using the Clustal colour scheme (http://www.jalview.org/help/html/colourSchemes/clustal.html). The amino acid co-ordinates of each TRP domain are listed on the right. The TPR consensus sequence is shown above. Accession numbers are WR F12 (AAO89330), WR E2 (YP_232940) and WR A36 (YP_233041).

F12 interacts with VACV proteins A36 [56] and E2 [55], and A36 can also interact *in vitro* with KLC TPRs via aa 81–111 [21]. Since TPRs mediate protein interactions [27], we wondered if A36 or E2 also contained TPRs. Surprisingly, examination of these proteins identified three TPR-like sequences in A36 and fifteen TPRs in E2 (72% of E2) (Figure 2). These observations suggested the association of A36 and E2 with F12 might be via TPR∶TPR interactions, and TPR∶TPR interactions might also facilitate A36 interaction with KLC. Consistent with the former hypothesis, F12 and A36 interact via residues 351–458 of F12, and 91–111 of A36, which overlap the TPRs [56].

**Figure 3:**
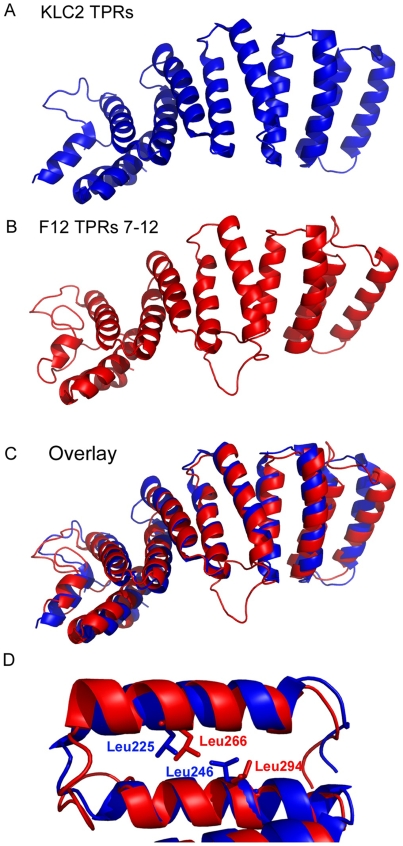
Structural model of F12 TPRs. The three-dimensional structure of the KLC2 TPRs (A) compares favourably with the model of the F12 TPR domains 7–12 (B) illustrated by an overlay of the two structures (C). (D) KLC2 TPR 1 is overlaid with F12 TPR 6 illustrating the spatial conservation of key hydrophobic aa side-chains responsible for holding together the two alpha helices that comprise a single TPR domain.

Based on the x-ray crystallographic structure of the KLC2 6 TPRs (pdb ID∶3CEQ), and its alignment with the F12 protein (Figure 1), a structural model of F12 TPRs 7–12 was built using the program MODELLER (Figure 3). This model satisfied all geometric and stereochemical constraints and the overall fold of the F12 TPRs 7–12 was remarkably similar to that of KLC2 TPRs (Figure 3A, B). Superimposition of KLC2 and F12 showed a very close match with variation restricted to loops connecting adjacent TPRs (Figure 3C). Importantly, the key hydrophobic residues that mediate hydrophobic interactions between the 2 alpha helices of the TPR fold, which are the hallmark of TPR domains, are structurally conserved between F12 and KLC2 (Figure 3D). Similar results were found for the F12 TPRs 1–6 (data not shown). Overall, analysis of the F12 primary sequence and predicted tertiary structure provides strong evidence of a TPR-containing protein with structural similarity to KLC.

### The entire F12 protein is required for IEV motility

**Figure 4:**
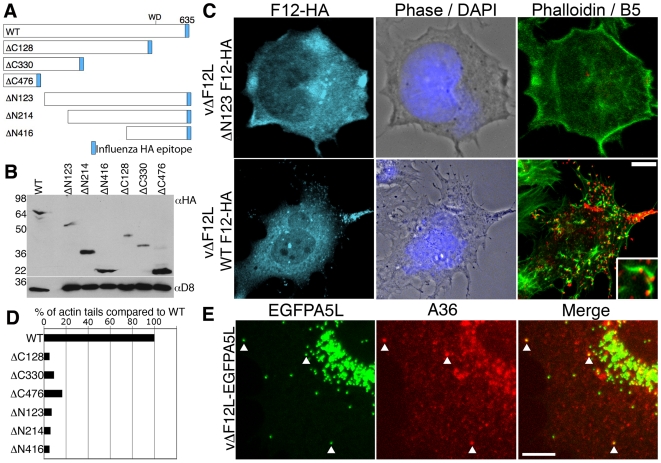
Full length F12 is required for IEV transport. (A) Illustration of HA-tagged F12 mutant proteins analysed. (B) HeLa cells were infected with vΔF12L at 5 pfu/cell and were transfected with plasmids encoding F12-HA at 2 h post infection. After 10 h cells were lysed and lysates were immunoblotted with anti-HA mAb. The blot was stripped and reprobed with anti-D8 mAb (a VACV protein) to confirm the equivalence of loading. (C, D) Rescue assay demonstrating that the full length F12-HA protein is required for the formation of cell surface virions and actin tails. HeLa cells were infected with vΔF12L-EGFPA5L at 5 pfu/cell for 2 h and then were transfected with plasmids expressing F12 for 10 h. Cells were stained with anti-B5 mAb (red) prior to fixation and staining with anti-HA mAb (cyan) and phalloidin (green). Inset shows B5-positive virus-tipped actin tails. Loss of surface B5 labelling and actin tail formation in cells transfected with the F12-HA truncations demonstrated virions have not reached the cell surface. Actin tails were counted and data were expressed as the percentage of actin tails formed by each mutant compared to WT. Data are from a single experiment that is representative of three. (E) A36 is localised to vΔF12L IEV. Cells infected as in (C) were permeabilised and stained for A36 (red) or visualized for EGFP. The merged image shows A36-positive IEV particles (arrows). Scale bars C = 10 µm, E = 5 µm.

In previous studies, deletion of the *F12L* gene showed the protein is not required for IEV formation but is needed for IEV transport to the cell periphery and CEV formation [18],[19]. To investigate which domains of F12 are needed for function, we generated a panel of N- and C-terminal truncations of F12-HA (Figure 4A) and cloned these into the pSEL vector downstream of a VACV promoter [57]. These *F12L* alleles were transfected into cells infected with vΔF12L and rescue of IEV transport to the cell surface was measured by first staining live cells with anti-B5 mAb for counting CEV, and second by staining permeabilised cells with phalloidin and counting virus-tipped actin tails [58]. Immunoblotting lysates of HeLa cells infected with the deletion F12 virus and transfected with the F12-HA mutant alleles confirmed the mutants expressed stable proteins of the expected size (Figure 4B).

**Figure 5:**
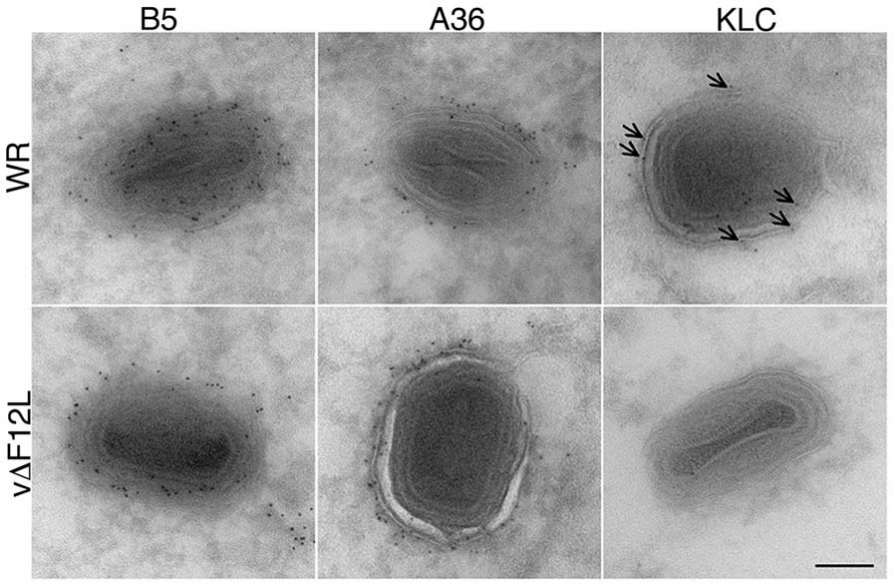
Cryoimmunoelectron microscopy showing F12 is needed for KLC, but not A36, recruitment to IEV. Cryoimmunoelectron microscopy of ultrathin sections of HeLa cells infected with VACV WR (top) or vΔF12L (bottom) and stained for B5, A36 or KLC with mAbs followed by 6-nm gold particles. Note B5 and A36 are present on VACV WR and vΔF12L IEV, but KLC is recruited to VACV WR IEV (arrows) but not to vΔF12L IEV. Scale bar = 100 nm.

In cells infected with vΔF12L, IEV particles were located in the centre of the cell, and CEV and actin tails were not present [18],[19]. In contrast, when full length F12-HA was expressed in these cells, it localised to structures in the cytosol and cell periphery typical of IEV (Figure 4C, cyan) and there were numerous B5 positive CEV (Figure 4C, red) and actin tails (Figure 4C, green; D). In comparison, all the F12 mutants failed to induce IEV dispersal to the cell periphery and did not generate CEV or actin tails (Figures 4C, D, S4). Importantly, the failure of vΔF12L IEV to move to the cell surface was not due to the lack of recruitment of A36 to virions, because confocal microscopy of vΔF12 IEV particles tagged with EGFP (vΔF12L-EGFPA5L) [19],[59] (Figure 4E) and cryoimmunoelectron microscopy (Figure 5) showed A36 was present on vΔF12L IEV. Moreover, A36 is non-essential for IEV transport as vΔA36R IEV are transported on microtubules [19].

### F12 is required for kinesin-1 recruitment

The important role of F12 in IEV movement suggests the protein may function in kinesin recruitment and/or activation [19]. To investigate this, the localisation of F12-HA and kinesin-1 was determined in vF12LHA-infected cells. Cells were processed for immunofluorescent microscopy after staining with a rat mAb against the HA tag [18] and with antiserum against KHC [60] (Figure S5). In mock-infected HeLa cells no specific anti-HA signal was detected and KHC was localised to structures in the cytoplasm as reported [60] (Figure S5, top row of panels). However, in infected cells KHC was predominantly located at the cell periphery and in punctate structures throughout the cell (Figure S5, bottom row, green), and a similar distribution was observed for F12-HA (Figure S5, bottom row, red). The merged image (Figure S5, bottom row, merge) showed a high degree of co-localisation, indicating kinesin-1 is recruited to F12-HA positive IEV particles in the cell periphery. The coincidence of F12-HA and kinesin-1 on IEV are consistent with F12 contributing to recruitment and retention of kinesin-1 allowing efficient transport.

**Figure 6:**
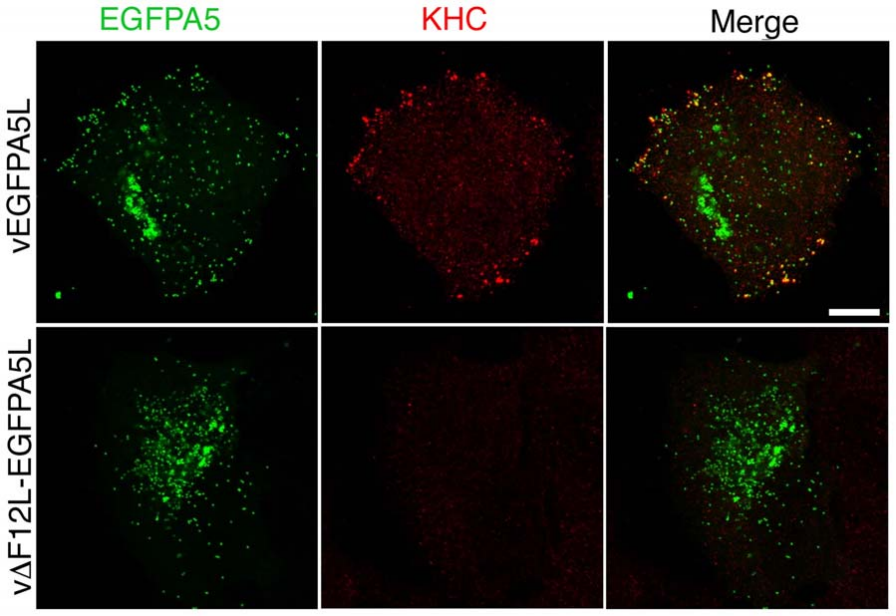
F12 is required for KHC recruitment. HeLa cells were infected with vEGFPA5L (top) or vΔF12-EGFPA5L (bottom) at 5 pfu/cell for 12 h. Cells were then fixed, permeabilised and stained for KHC (red) and visualized for EGFP (green). The merged image shows KHC recruitment to IEV particles at the periphery of the cell in vEGFPA5L-infected cells. Note KHC recruitment to IEV particles does not occur in cells infected vΔF12-EGFPA5L. Scale bar = 10 µm.

**Figure 7:**
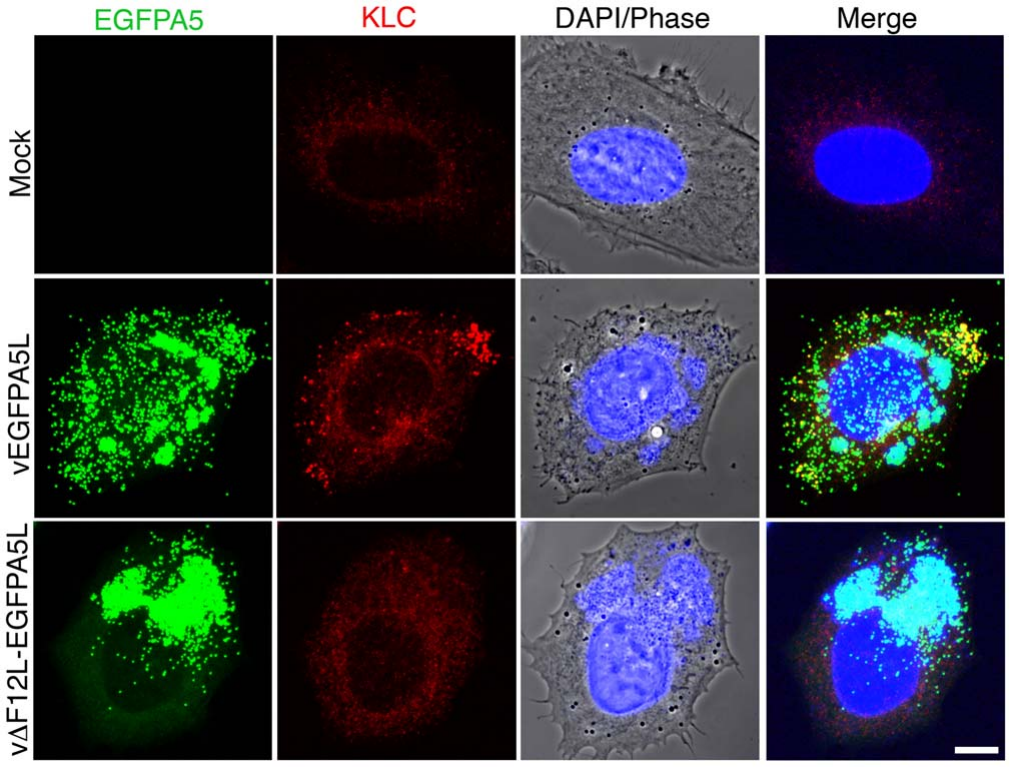
F12 is required for KLC recruitment. HeLa cells were either mock-infected or infected at 5 pfu/cell with vEGFPA5L or vΔF12-EGFPA5L. At 12 h p.i. cells were fixed, permeabilised and stained for KLC (red), DAPI (blue) or visualized for EGFP (green). The merged image is shown on the right. Note KLC is recruited to IEV particles at the periphery of the cell in vEGFPA5L-infected cells, but not in cells infected with vΔF12-EGFPA5L. Scale bar = 10 µm.

The requirement of F12 for IEV motility is consistent with the suggested role in kinesin-1 recruitment and/or function. This was investigated further by confocal microscopy using viruses that have EGFP fused to the A5 core protein, and do or do not express F12, vEGFPA5L [61] and vΔF12L-EGFPA5L [19]. In cells infected with vEGFPA5L, kinesin-1 co-localised with virions in the cell periphery, with similar results observed for the KHC and KLC (Figures 6 and 7). In contrast, after infection with vΔF12L-EGFPA5L, kinesin-1 was not recruited to IEV and remained diffuse in the cytoplasm (Figures 6 and 7). Cryoimmunoelectron microscopy confirmed KLC was recruited to wild type WR IEV and showed it was absent from vΔF12L IEV (Figure 5). The recruitment of kinesin-1 to wild type IEV but not vΔF12L IEV despite the presence of A36 in both cases, demonstrates F12 is needed for kinesin recruitment and A36 alone is not sufficient.

### F12 and E2 are not required for IEV formation

**Figure 8:**
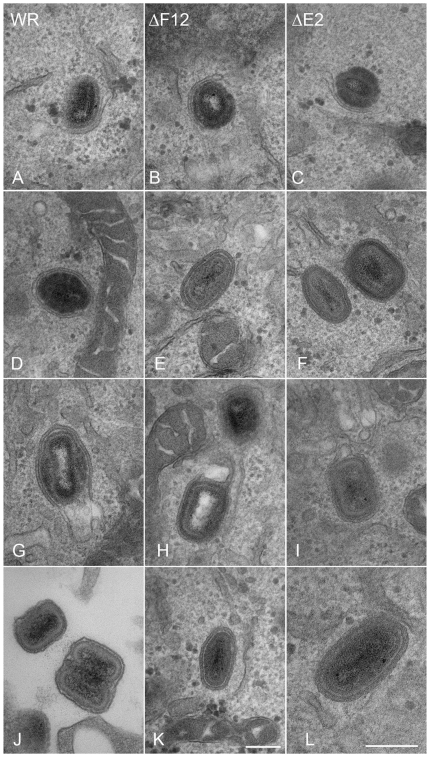
F12 is not required for IEV formation. HeLa cells were infected at 5 pfu/cell with WR (A, D, G, J), vΔF12L (ΔF12) (B, E, H, K) or vVAC-BACΔE2L (ΔE2) (C, F, I, L) for 12 h. Cells were then processed for thin 70-nm serial-section analysis using conventional electron microscopy. Images show virions at different stages of morphogenesis: (A–C) IMV association with wrapping membranes, (D–F, K and L) complete IEV, (G–I) complete IEV in which part of the wrapping membrane is not tightly associated with the virions, and (J) CEV/EEV formed by WR. Micrographs A–J & L are shown at the same magnification. Scale bars = 200 nM.

**Figure 9:**
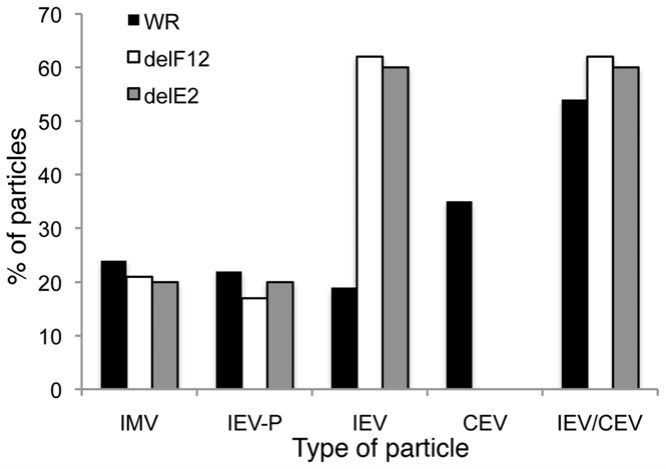
Enumeration of types of virion produced by WR, vΔF12L and vΔE2L. HeLa cells were infected at 5 pfu/cell with WR, vΔF12L or vVAC-BACΔE2L for 12 h. Cells were then fixed and processed for conventional electron microscopy. Viral particles (*n* = 100) were counted in thin sections and the percentage of IMV, partially wrapped IEV, completely wrapped IEV and CEV were determined and are expressed as a percentage of total virions. Note: i) loss of F12 and E2 caused no difference in the number of partially wrapped IEV relative to wild type but there were three times as many IEV in cells infected with deletion viruses; ii) No CEV were seen for the deletion mutants but the sum of IEV/CEV were equivalent for mutants compared to wild type.

In the first paper describing E2 and its role in the VACV life cycle Domi et al [54] reported that without E2 there was a 100-fold reduction in extracellular virus. Electron microscopy and analysis of several hundred virions in infected cells, showed there were comparable numbers of immature virions, IMVs and IEVs but the amount of CEV/EEV was reduced dramatically [54]. In a later paper Dodding et al [55] examined morphogenesis of vΔE2L and vΔF12L using electron microscopy of thick sections of infected cells and concluded that there was a defect in wrapping of IMVs to form IEVs in both cases [55]. In the light of these conflicting reports, we used thin section transmission electron microscopy, tilt series analysis and serial section analysis to re-investigate the roles of F12 and E2 in IEV morphogenesis. Thin section analysis of HeLa cells infected with WR, vΔE2L and vΔF12L showed the usual stages of IEV morphogenesis for all viruses, namely IMV association with wrapping membrane (Figure 8A–C), IEV (Figure 8D-F, K and L), and IEV with loose wrapping membrane (Figure 8G–I). With wild type (WR) virus there were numerous CEV/EEV particles outside the cell (Figure 8J), but these were not seen with either vΔE2L or vΔF12L. To determine if the proportions of the different virions varied between wild type and mutants, viral particles were counted in thin sections and the percentage of IMV, partially wrapped IEV, completely wrapped IEV and CEV was determined (Figure 9). Loss of F12 and E2 cause no difference in the number of IMV, or partially wrapped IEV relative to wild type, but there was a three-fold increase in IEV with vΔE2L and vΔF12L. The increase in IEV is likely attributable to inefficient transport of vΔF12L and vΔE2L IEV to the plasma membrane, consistent with the lack of CEV formed by these viruses. Notably the sum of IEV and CEV for each mutant was equivalent to wild type.

**Figure 10:**
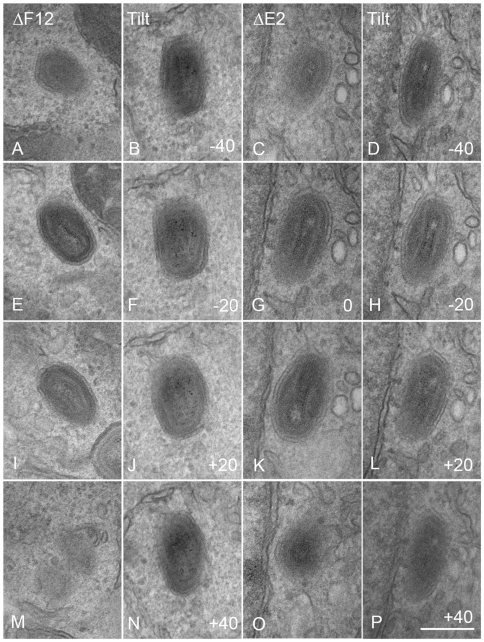
Serial section and tilt series electron microscopy showing vΔF12L and vΔE2L IEV are fully wrapped. Cells were infected and processed for thin 90-nm serial section electron microscopy as described in Figure 8. Serial section analysis of vΔF12L (ΔF12, A, E, I, M) and or vVAC-BACΔE2L (ΔE2, C, G, K, O). Tilt series analysis of vΔF12L (B, F, J, N) (derived from a different IEV) or vΔ vVAC-BACΔE2L (D, H, L, P) IEV tilted the indicated degree in the electron beam. For vVAC-BACΔE2L, the section shown in (G) was tilted through 20° increments. Note the continuity of the double membrane forming the IEV particles. All micrographs are shown at the same magnification. Bar, 200 nm.

By counting many virus particles (*n* = 100) for each virus in our study, it was unlikely that any defect in IEV wrapping would have been missed in the thin sections examined. Similarly, Domi et al [54] examined several hundred vΔE2L virions and found numerous normal IEV. However, to further investigate IEV wrapping we analysed serial sections of vΔF12L (Figure 10, A, E, I, M) and vΔE2L IEV (Figure 10, C, G, K, O). In addition, we undertook tilt series analysis to bring membranes perpendicular to the electron beam, and therefore in sharp focus, for vΔF12L (Figure 10, B, F, J, N) and vΔE2L IEV (Figure 10, D, H, L, P). For both viruses this demonstrated the presence of many completely wrapped IEV with double membranes surrounding IMV (Figure 10). Moreover, in cases where membranes were not in sharp focus in one plane, that might suggest incomplete wrapping, tilting the section ±50° brought these double membranes into sharp focus as they became perpendicular to the electron beam, see full tilt series animation of vΔF12L in Video S1. We conclude that vΔE2L and vΔF12L form IEV normally and are indistinguishable from wild type. The lack of vΔF12L IEV transport to the plasma membrane is therefore not due to a morphological defect but to failure to recruit kinesin-1.

### Kinesin-1 is recruited via a kinesin binding sequence (WD motif) in F12

**Figure 11:**
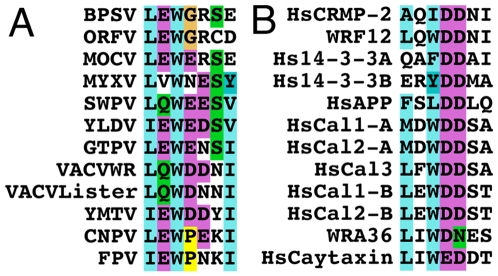
Conservation of the WD kinesin-1 binding site. (A) Alignment of the WD kinesin-1 binding site in F12 orthologues in chordopoxviruses with the consensus (L-E-W-E-E/D-S-I) sequence shown below. (B) Alignment of the WD kinesin-binding site in other protein reported to bind KLC with the consensus (L-E/D-W-D-D-S-A) sequence shown below. Overall these sequences suggest the WD KBS conforms to the sequence L-E/D-W/ϕ-D/E-D/E-S/N (Where ϕ is another aromatic residue). The Human 14-3-3 protein and calsyntenin-1 and calsyntenin-2 contain two WD motifs shown as A and B in the alignment. Accession numbers are VACV WR F12 (AAO89330), Lister F12 (DQ121394.1) Myxoma virus (MYXV: NP_051735), Yaba-like disease virus (YLDV: NP_073411), Swinepox virus (SWPV: NP_570184.1), Molluscum contagiosum virus (MOCV: MC019L), Goatpox virus (GTPV: YP_001293217.1), Bovine papular stomatitis virus (BPSV: NP_957919.1), Yaba monkey tumor virus (YMTV: NP_938282.1), Orf virus (ORFV: AAR98105.1), Canarypox virus (CNPV: NP_955159.1), Fowlpox virus (FPV: NP_039072.1), VACV WR A36 (YP_233041), Human collapsin response mediator protein-2 (HsCRMP-2, Q16555), Human calsyntenin-1 (HsCal1: CLSTN1), Human calsyntenin-2 (HsCal2: CLSTN2), Human calsyntenin-3 (HsCal3: CLSTN3), Human 14-3-3 protein (Hs14-3-3: BAG35533), Human caytaxin (HsCaytaxin: NM_033064) and Human amyloid precursor protein (HsAPP: 351 APP).

Data presented show that F12 is required for kinesin-1 recruitment and IEV transport. To investigate further how F12 might recruit kinesin-1 we searched for known kinesin-1 binding sequences (KBS) within the F12 protein. A recent study identified two copies of a KBS in the neuronal transmembrane protein calsyntenin/alcadein conforming to the consensus sequence L/M–E/D–W–D–D–S, termed the WD motif, after the conserved tryptophan and aspartic acid residues [30],[62]. A WD motif is also present in the Cayman ataxia protein caytaxin, which also binds KLC [36]. The calsyntenin study also identified a putative KBS within protein A36 (L–I–W–D–N–E, residues 95 to 100) [30]. Inspection of the WR F12 protein revealed a similar motif L–Q–W–D–D–N at residues 534 to 539 (underlined in Figure 1) and further analysis showed this WD motif is conserved in F12 orthologues from chordopoxviruses of diverse genera with the consensus sequence L-E-W-E-E/D-S (Figure 11A). In VACV strains Ankara, Lister and Copenhagen this sequence is L–Q–W–D–N–N and the D to N substitution makes the sequence more similar to the WD motif in A36.

A search for the WD motif in other cellular proteins identified similar sequences in the collapsin response mediator protein-2, 14-3-3 protein and amyloid precursor protein with the consensus sequence (L-E/D-W-D-D-S) (Figure 11B). These proteins are known to bind KLC TPRs. Overall this analysis suggests the WD kinesin-1 binding motif conforms to the sequence L-E/D-W/ϕ-D/E-D/E-S/N (where ϕ is an aromatic residue other than tryptophan).

**Figure 12:**
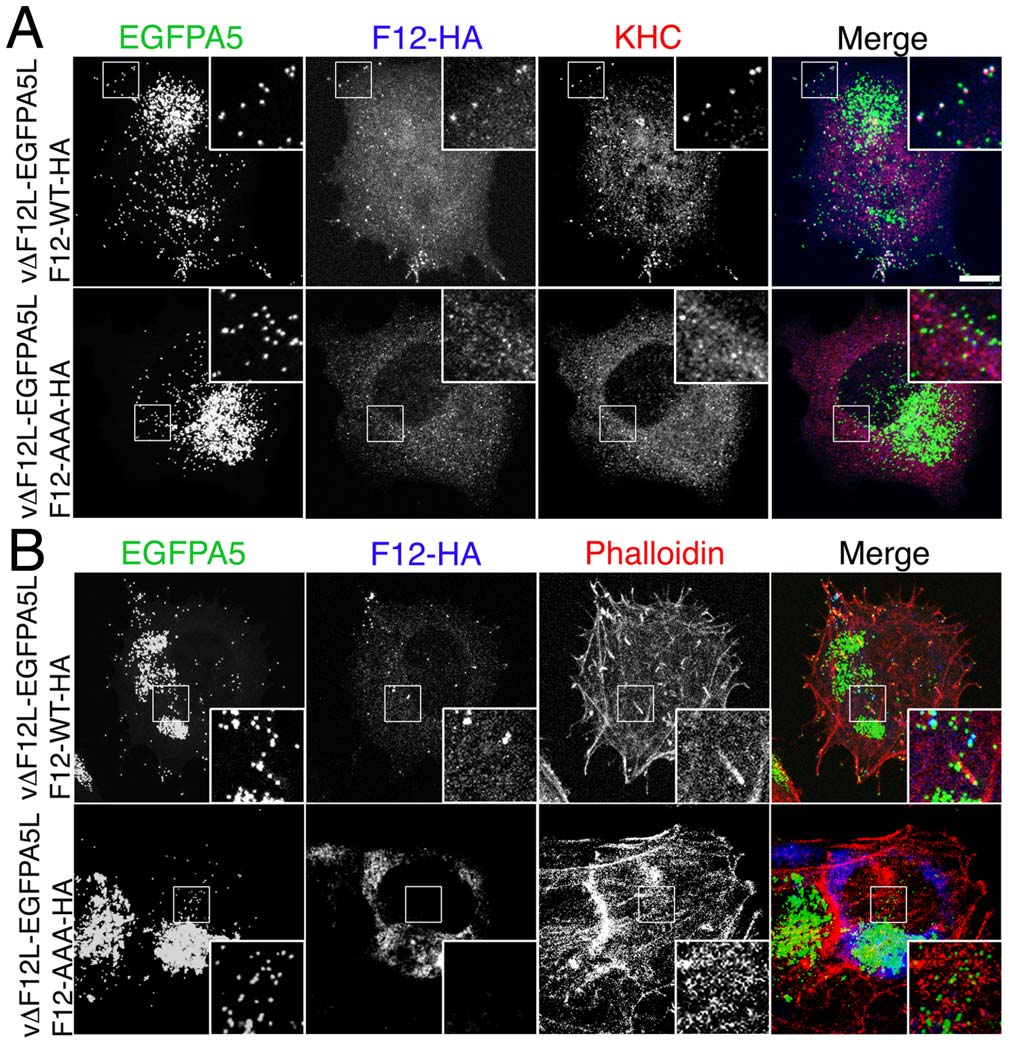
The F12 WD motif is needed for kinesin-1 recruitment and IEV transport. (A) HeLa cells were infected at 5 pfu/cell with vΔF12L-EGFPA5L for 2 h. Cells were then transfected with plasmids expressing WT or mutant F12-HA, and 10 h later were stained with mAbs against HA (blue) or KHC (red), or visualized for EGFP. The overlay is shown in the Merge image. In cells transfected with F12-WT-HA the particles are widely distributed and can recruit both F12-HA and KHC (white in merged image). In cells transfected with the mutant F12-AAA-HA the particles remain centrally localized, and are unable to recruit either F12-HA or KHC. (B) Cells were infected and transfected as in (A) and then stained for HA (blue), and phalloidin (red) to identify actin tails. Top panels shows F12-WT-HA is targeted to widely distributed particles and actin tails are formed at the plasma membrane. Note virions on actin tails are negative for F12L-HA. Bottom panel show absence of F12 recruitment and lack of actin tail formation. Insets show ×2.5 enlargements of the boxed regions. Scale bar = 10 µm.

**Figure 13:**
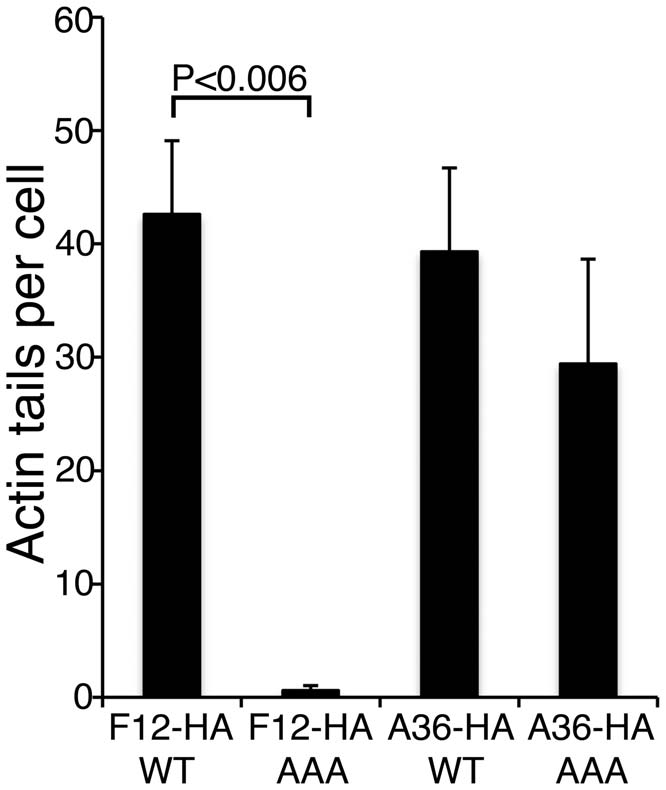
The F12 WD motif is needed for kinesin-1 recruitment. Quantification of actin tail formation by analysis of data shown in Figures 12 and S8. Data are the means +/− SEM of triplicate experiments. Note mutation of F12 WD motif inhibits actin tail formation, whereas mutation of the A36 WD motif does not.

The roles of the F12 WD motif in kinesin-1 recruitment and IEV transport were investigated by mutation of WDD to AAA, a mutation that abrogates kinesin-1 binding by calsyntenin/alcadein [30]. Confocal microscopy demonstrated that wild-type F12-HA and KHC were targeted to EGFP positive IEV in the cell periphery (Figure 12A) and that actin tails were formed from the surface of these cells (Figures 12B & 13). In contrast, mutation of the WD motif blocked the recruitment of F12-HA and KHC to IEVs (Figure 12A), and most virions remain centrally localised with very few particles moving to the cellular periphery or generating actin tails (Figure 12B). This loss of F12 function was not due to instability of the F12 mutant protein because immunoblotting showed that F12-HA WT and F12-HA AAA were present at similar levels (Figure S6). Mutation of the F12 WD motif reduced cell surface actin tails by 98% (Figure 12B and 13), demonstrating that the WD motif is necessary for IEV recruitment and transport. However, it is not sufficient because F12 mutants retaining the WD motif, but lacking other parts of the protein do not enable virion motility (Figure 4D, S4).

In parallel we also investigated the role of the WD motif (L–I–W–D–N–E) within protein A36 reported in the calsyntenin study [30]. Bioinformatic analysis found the sequence is conserved in A36 orthologues from chordopoxviruses and an alignment of these sequences is shown in Figure S7. Similar mutational analysis of the A36-HA protein showed the A36-AAA-HA protein is stable (Figure S6), recruited to IEV (Figure S8A) and enables IEV transport to the cell surface where actin tails were formed at 69% of wild-type level (Figure 13, S8B). This small defect in IEV transport/actin tail formation is comparable to that observed following deletion of the entire *A36R* gene, where IEV still move on microtubules and reach the cell surface at 60% of wild type levels [19].

We also identified another putative KBS in VACV A36 (S-D-W-E-D-H, residues 62–67) that only conforms partially to the WD consensus sequence. Although this motif is not conserved outside of the orthopoxvirus A36 orthologues, we undertook a similar mutagenic analysis. Mutation of the WED to AAA alone, or in addition to the WDN to AAA at residues 97–99 to form a double mutant, gave no phenotype (data not shown). This mutagenic analysis demonstrates the WD motif present within F12, but not those in A36, is critical for microtubule-mediated virion transport.

## Discussion

How motor proteins select and bind intracellular cargo is an important biological question. The importance of intracellular transport is highlighted by the fact that mutations in motors lead to genetic diseases such as amyotrophic lateral sclerosis, paraplegia and Griscelli syndrome type 1 [63]. Kinesin-1, the archetypal member of the kinesin superfamily, drives the transport of diverse cellular cargos such as neuronal vesicles, secretory vesicles, endocytic structures, mitochondria and mRNP particles and has been implicated in the dynamics of the endoplasmic reticulum (ER) and Golgi apparatus [64]. Kinesin-1 is involved in the intracellular motility of pathogens and in the development of neurodegenerative disorders including Alzheimer's disease. Hence, studying factors coordinating kinesin-1 cargo binding is of biological and clinical importance.

This paper concerns how VACV exploits kinesin-1 for transport of virions to the cell periphery. We have discovered that VACV transport protein F12 is structurally related to KLC and shares with KLC a similar size, pI, hydrophobicity profile and TPRs. Bioinformatic analysis and structural modelling of F12 TPRs 7–12 with the recently described crystal structure of KLC2 TPRs showed a striking structural similarity. A close relationship was also found for F12 TPRs 1–6, suggesting that F12 might have evolved by duplication of the TPR domain of KLC accompanied by deletion of the N-terminal heptad repeats. Mutagenesis of the F12 protein showed that deletion of these TPRs from either terminus abrogated F12 function, implying an important role for TPRs. Although these mutant proteins were reasonably stable in virus-infected cells, we cannot exclude the possibility that the loss of function was due to aberrant folding of truncated proteins. F12 also contains a WD kinesin-1 binding motif that is found in several cellular cargo proteins and mutagenesis demonstrated that the F12 WD motif is critical for recruitment of kinesin-1 and IEV transport. A model consonant with these observations is that F12 binds cargo (virions) via TPR∶TPR interactions with E2 (or A36, although A36 is not essential for IEV binding to microtubules and transport), and that it uses the WD motif to attach to KLC and thereby the kinesin-1 motor. Thus F12 functions as a bridge between microtubule motor and virus particle. Recently, the Cayman ataxia protein caytaxin was proposed to have an analogous function and shown to bind to KLC TPRs via a WD motif and mediate transport of cargos, such as mitochondria, by specific binding to these structures [36].

This example of VACV mimicry of a host cell protein adds to a growing list. Other examples include K3, a mimic of eukaryotic initiation factor 2α [65], A44, a 3β-hydroxysteroid dehydrogenase [66], B15, a soluble interleukin-1β receptor [67] and proteins N1, B14 and A52 that have Bcl-2 structures and inhibit apoptosis or NF-κB signaling [68]–[70]. However, the F12 protein represents the first example of a VACV protein mimicking a host cell motor protein and to our knowledge is the only viral KLC structural mimic described.

### TPRs in F12 and other VACV IEV proteins

In addition to finding TPRs in F12, we report that TPRs are also present in proteins E2 and A36 which, like F12, have a role in IEV formation or transport. TPRs mediate protein-protein interactions and therefore the presence of TPRs in several VACV proteins that associate with each other suggests that these interactions might be via TPR∶TPR contacts. Consistent with this, F12 and A36 interact through residues 351–458 of F12, and 91–111 of A36, which overlap the TPRs of both proteins [56]. The region of A36 needed to bind to KLC *in vitro* (aa 81–111) [21] also overlaps the A36 TPRs reported here. A recently described function of the KLC TPRs is in the activation of the KHC upon cargo binding [71] and it is possible that F12 may have a similar effect.

### Regulation of kinesin-1 recruitment to IEV

Previously, immunoelectron microscopy demonstrated that F12 is associated transiently with VACV during morphogenesis and egress and is present only on IEV, but not IV, IMV or CEV [18]. Confocal microscopy also showed that F12 co-localised with IEV particles but was absent from CEV particles associated with actin tails at the cell surface [18],[55]. Furthermore, although IEV were formed in the absence of F12, they were not transported to the cell periphery and consequently morphogenesis was arrested prior to the formation of CEV and actin tails [18],[19]. Recently, Dodding et al [55] challenged the view that IEV are formed without F12 or E2. Using thick sections of infected cells they concluded that only incompletely wrapped IEV were formed, but we wondered if the resolution of the images presented was sufficient to allow such a conclusion. Previously, high resolution images of thin sections of vΔF12L-infected cells showed multiple IEVs with continuous outer membranes [18]. Similar images are presented here and these were indistinguishable from wild type. In addition, we undertook serial section and tilt series analyses that showed both F12 and E2 deletion viruses wrap normally. Therefore, the transport defect observed in these mutants is not caused by a failure to form IEV. These observations are consistent with a role for F12 in IEV transport on microtubules to the cell periphery. To investigate this, we showed here that F12 and kinesin-1 are both present on IEV particles and, in the absence of F12, kinesin-1 was not recruited to IEV. Notably, immunoelectron microscopy and confocal microscopy demonstrated that A36 was still recruited to IEV in the absence of F12, showing A36 is not sufficient for kinesin-1 recruitment.

To investigate which domains of F12 are needed for kinesin-1 recruitment and IEV transport, we utilised a rescue assay in which cells are infected with a mutant virus lacking the *F12L* gene and then are transfected with wild type F12 tagged with HA (F12-HA), or with mutants thereof. F12 function was assessed by kinesin-1 recruitment, dispersal of virions to the cell periphery, CEV formation and the production of actin tails from the cell surface. This showed that all F12 mutants tested with deletions from either terminus were non-functional, and mutation of the WDD motif to AAA also inhibited both kinesin-1 recruitment and IEV transport. A direct F12∶kinesin-1 interaction was not detected in previous yeast two hybrid screens [21]. However, this does not preclude a direct interaction because F12 may act with additional factors on the IEV surface to recruit kinesin-1. Also, the interaction between A36 and KLC seen *in vitro* has still to be demonstrated within infected cells.

An earlier study describing the WD kinesin-1 binding sequence in the neuronal transmembrane protein calsyntenin/alcadein reported that two WD motifs were needed for function, and these authors reported a WD motif in A36 [30]. In contrast to the situation with F12, we show here that mutation of the A36 WD motif did not inhibit kinesin-1 recruitment, IEV transport, and CEV and actin tail formation. However, it was clear that these processes were slightly less efficient with the A36 WDN to AAA mutation, and actin tails were produced at about two thirds the level of wild type. So the WD motif in F12 and A36 each play a role, but the requirement for the F12 WD motif is much more profound and the A36 plays a minor supporting role (Figure 13). These observations are similar to those resulting from deletion of the entire A36 protein, which caused a decrease in the efficiency of IEV transport and CEV formation (60% of wild type levels at 24 h p.i.) [19]. The results from mutation of the WD motifs in A36 and F12 have similarity with calsynentin/alcadein where mutation of either WD motif reduced binding to KLC1 to 30% compared with wild type calsyntenin/alcadein, and when both motifs were mutated only 3% of the WT binding was achieved [30]. This indicates that only one calsyntenin/alcadein KBS is insufficient to mediate efficient interaction and that both binding sites are needed for optimal binding to KLC. The bipartite nature of the WD signal in calsyntenin/alcadein may explain why two VACV proteins have this motif and both are needed for optimal efficiency. The more profound requirement for F12 may be a mechanism used by VACV to ensure kinesin-1 is recruited efficiently to only fully formed IEV containing both F12 and A36, and not just A36 containing membranes that are used to enwrap IMVs to form IEVs. This may provide a quality control checkpoint enhancing transport of fully formed IEV rather than precursors to the cell surface. In this paper we also identify WD motifs in other proteins that bind directly to the KLC raising the possibility that the use of co-receptors may be a common mechanism for kinesin-1 attachment and regulation of cargo transport. Indeed, co-operative mechanisms of cargo binding to the KLC TPR are currently being studied [39].

Previously, protein A36 was described as essential for kinesin-1 recruitment to IEV and for IEV transport [17]. However, while it is evident that A36 does influence IEV transport, several lines of evidence show it is not critical for this. First, live video microscopy showed that IEV lacking A36 move at speeds consistent with kinesin-1 transport, this movement was inhibited by microtubule depolymerising drugs, and A36 null virions are dispersed to the cell periphery [19]. Second, several reports using confocal or electron microscopy have shown CEV are formed in the absence of A36 [14],[18],[19],[51],[52]. Third, it is shown here that in the presence of A36, but absence of F12, kinesin-1 is not recruited to IEV, indicating A36 is not sufficient for kinesin-1 recruitment. As mentioned above, this may have an important consequence in preventing kinesin-1 recruitment to A36 containing membranes such as the ER, through which it trafficks after co-translational insertion [72], or the plasma membrane where it is deposited during virus egress from the cell [72]. Only fully formed IEV that contain F12 require kinesin-1 recruitment and transport. It is possible that Rietdorf et al [17] concluded that A36 is essential for kinesin recruitment and IEV movement because their investigation was conducted only early during infection. Herrero-Martinez et al [19] showed that the transport of vΔA36R IEV is delayed compared to wild type, but by 24 h p.i. the level of CEV reach 60% of wild type. We conclude that, although A36 plays a minor role, F12 is the more important protein for kinesin-1 based transport of IEV.

In summary, VACV F12 is a KLC structural mimic containing TPRs and a KBS, and VACV proteins A36 and E2 also contain TPRs that may mediate protein∶protein interactions. In addition, we show that the calsyntenin/alcadein WD motif for binding kinesin-1 is conserved in several other cellular kinesin-binding proteins and in VACV proteins F12 and A36. The F12 WD motif is critical for kinesin-1 recruitment and virion transport to the cell surface, whereas the A36 WD motif plays a supporting role. This report advances our understanding of VACV transport to the cell surface and illustrates how poxviruses use molecular mimicry for subversion of host cell biology.

## Materials and Methods

### Cells and viruses

HeLa cells were grown in minimum essential medium (MEM) supplemented with 10% heat-inactivated foetal bovine serum (Gibco). The viruses used in this study were vaccinia virus (VACV) strain Western Reserve (WR) and derivative strains vΔF12L, F12-HA [20], vΔA36R [50], vEGFPA5L [61], vΔF12L-EGFPA5L and vΔA36R-EGFPA5L [19]. Cells were infected at 5 plaque forming units (PFU)/cell. For confocal microscopy cells were fixed at 12 h p.i.

### DNA manipulation

PCR was performed with Pfu polymerase (Stratagene). The fidelity of the cloned fragments was verified by DNA sequencing. Mutants of F12 with N- or C-terminal truncations were constructed by PCR using VACV WR or vF12L-HA genomic DNA as template for the C- or N- terminal mutants, respectively. For the N-terminal mutants, the oligonucleotides 5′-GAACAAGCTTGCCATCATGGGTATCGAGAATACAGATTCCAT-3′ (F12-ΔN123); 5′- GAACAAGCTTGCCATCATGTCCTCGTTAGATCAAACACA-3′ (F12-ΔN214) and 5′- GAACAAGCTTGCCATCATGGGAATGGGGATGTATTATCCTA-3′ (F12-ΔN416) containing a *Hin*dIII restriction site (underlined) and 5′-GAACGGATCCTTAAGCGTAATCAGGCACG-3′ (F12-HA R1) containing a *Bam*HI restriction site (underlined) were used. The PCR product was digested with *Hin*dIII and *Bam*HI and ligated into *Hin*dIII and *Bam*HI- restricted pSEL, a pBluescipt vector containing the VACV synthetic early/late (E/L) [58] to form the pSE/L-F12-ΔN truncated mutants. For the C-terminal mutants, the oligonucleotides 5′-GAACGGATCCTTAGGTAGTATTGTCATCATCGTGAT-3′ (F12-ΔC128); 5′-GAACGGATCCTTAGATTTTCCATCCACAATTATTG-3′ (F12-ΔC330) and 5′-GAACGGATCCTTAATACATCTGTTTCCTATAATCGTT-3′ (F12-ΔC476) containing a *Bam*HI restriction site (underlined) and 5′-GAACAAGCTTATGTTAAACAGGGTACAAATCTTG-3′ (F12-F1) containing a *Hin*dIII restriction site (underlined) were used. To generate A36-HA primers A36-F *Hin*dIII *Bam*HI, 5′-CCCAAGCTTATGATGCTGGTACCTCTTATCACGG-3′ and A36-R HA *Bam*HI, 5′- GGAGGATCCTTAAGCGTAATCTGGAACATCGTATGGGTACACCAATGATACGACCGATGATTC-3′ were used. The PCR fragments were digested with *Hin*dIII and *Bam*HI and ligated into *Hin*dIII- and *Bam*HI-digested pSEL to form the pSEL-F12-ΔC truncated mutants. The WD motifs within A36 and F12 were mutated to alanines (F12-HA WDD534AAA and A36-HA WDN95AAA) using the QuikChange Site-Directed Mutagenesis Kit (Stratagene) using pSEL-F12-HA and pSEL-A36-HA as template.

### Infection and transfection for ectopic protein expression

HeLa cells were seeded on sterile glass coverslips and infected with VACV. At 2 h p.i. cells were transfected with 1 µg of plasmid using Fugene 6 (Roche Diagnostics) following the manufacturer's instructions. At 12 h post-transfection the cells were harvested or fixed and processed for immunofluorescence as detailed below.

### Antibodies and cytochemical reagents

Antibodies used were mouse mAb 63–90 α-KLC (IgG1) [73], mouse mAb H2 α-KHC (IgG2b) [60], mouse mAb 6.3 α-A36 [72], rat mAb 19C2 α-B5 [13] directly conjugated to Alex546, mouse mAb AB1.1 α-D8 [50], mouse mAb L2 α-KLC (Abcam), mouse mAb HA.11 α-HA (Convance Research Products), rat high affinity α-HA mAb (Roche) and rabbit anti-HA (Sigma). Actin was visualized with phalloidin labelled with Alexa Fluor 488 and 647 (Molecular Probes).

### Triton X-114 extraction

Cells were partitioned using Triton X-114 as described previously [50].

### Statistical analysis

In each experiment the number of actin tails per cell were counted for 10 cells for each infection/transfection, and the experiment was performed three times. Data were analysed by the Student's *t*-test to test the significance of the results.

### Microscopy

Cells were infected with VACV for 12 h, washed in ice-cold phosphate buffered saline (PBS) and fixed in 4% paraformaldehyde (PFA) in 250 mM Hepes pH 7.4 for 10 min on ice, and then in 4 or 8% PFA at room temperature (RT) for 10–50 min. Cells were permeabilised in 0.1 TritonX-100 in PBS for 10 min at RT. Alternatively, cells were for permeabilised for 5 min at RT in PBS containing 0.1% saponin, and the detergent was retained in all subsequent buffers when processing saponin-permeabilised coverslips. Fixed cells were processed for immunofluorescence as described previously [18]. Confocal images were captured using a Zeiss Pascal or Zeiss 510 Meta confocal microscopes using Zeiss LSM software. Images were assembled using ImageJ (http://rsbweb.nih.gov/ij/) and Adobe Photoshop (Adobe Systems). Images shown are maximum projections of confocal Z stacks with accompanying phase transmission image. For conventional electron microscopy, at 12 h post infection cells were fixed in 0.5% glutaraldehyde-200 mM sodium cacodylate (pH 7.4) for 30 min, washed in 200 mM sodium cacodylate, and postfixed in 1% osmium tetroxide-1.5% potassium ferrocyanide for 60 min at room temperature. The cells were washed in water, incubated in 0.5% magnesium-uranyl acetate overnight at 4°C, washed again in water, dehydrated in ethanol, and embedded flat in Epon. Sections were cut parallel to the surface of the dish. Serial sections were collected onto slot grids, and lead citrate was added for contrast. Grids were viewed on a FEI Tecnai G2 transmission electron microscope. Images were acquired using a CCD camera. For cryoimmuno-electron microscopy primary antibodies were detected using antibodies for appropriate species then protein A-gold 6 nm conjugate.

### Bioinformatics

BLAST searches [74] were conducted at NCBI (http://www.ncbi.nlm.nih.gov/blast). Sequence alignments were performed using Clustal X [75] and Jalview [76] and analysed using GeneDoc (http://www.psc.edu/biomed/genedoc). The predicted TPR domains 6–12 of F12 were subjected to structural modelling based on the alignment with KLC2 (Figure 1) and the structure of KLC2 TPR region (pdb ID∶3CEQ). The MODELLER software [77] was used to create 30 potential models of this protein region and these were analyzed using the PROCHECK [78], WHAT IF [79] and Verify3D [80] algorithms to ensure the satisfaction of stereochemical constraints. The model with the lowest energy and fewest spatial violations was selected as the most accurate representation of this domain. Protein structure illustrations were generated with the PyMOL Molecular Graphics System (http://www.pymol.org).

## Supporting Information

Figure S1 Kyte-Doolittle hydropathy plots of the VACV WR F12 and *Homo sapiens* KLC1 (HsKLC1). Note VACV WR F12 and HsKLC are similar in length and exhibit a similar oscillating hydrophobicity profile.
(0.83 MB PDF)

Figure S2 F12 is a cytosolic protein. F12-HA TritonX-114 partitioning. HeLa cells were infected at 5 pfu/cell with vF12L-HA for 12 h. Cells were then collected, lysed and subjected to TritonX-114 partitioning (Materials and Methods). Proteins in the soluble aqueous (Aq) or membrane detergent (Det) fractions were resolved by SDS-PAGE and immunoblotted with mAbs against HA (F12-HA) (left panel), or VACV protein D8 (membrane protein) and GSK3β (cytosolic protein) (right panel). The positions of molecular size markers are indicated in kDa.
(0.47 MB PDF)

Figure S3 Alignment of F12 orthologues. F12 orthologues from different chordopoxvirus genera were aligned using Clustal-X and are displayed using the Clustal colour scheme (http://www.jalview.org/help/html/colourSchemes/clustal.html). The positions of TPRs numbered 1–14 are indicated above the alignment. The F12 TPRs that were aligned with KLC TPRs in Figure 1 are TPRs 6–10 and 12. Note the conservation of aromatic residues throughout the alignment and the lack of N-terminal heptad repeats, which mediate KHC binding by KLC. Abbreviations: BPSV, Bovine papular stomatitis virus; CNPV, canarypox virus; FWPV, fowlpox virus; GTPV, goatpox virus; MOCV, molluscum contagiosum; MYXV, myxoma virus; ORFV, orf virus; SWPV, swinepox virus; WR, VACV strain Western Reserve; YMTV, yaba monkey tumor virus.
(4.95 MB TIF)

Figure S4 Full length F12 protein is required for IEV transport to cell periphery. HeLa cells were infected at 5 pfu/cell with vΔF12L and at 2 h p.i. were transfected with plasmids expressing full length F12L-HA or the indicated F12 mutants. The cells were fixed at 12 h p.i., permeabilised and stained with mAb against HA (blue), or B5 (red), and phalloidin-Alexa Fluor 488 to label filamentous actin (green). Cells were then viewed by confocal microscopy. The insert shows B5-positive virus particles at the tips of actin tails in cells transfected with the WT F12. Scale bar = 10 µm.
(4.38 MB PDF)

Figure S5 F12 co-localises with kinesin-1. Confocal microscopy showing colocalisation of F12-HA and KHC. HeLa cells were mock-infected or infected with vF12L-HA at 5 pfu/cell. At 12 h p.i. cells were permeabilised and stained with α-HA (F12-HA, green) and α-KHC (red). The right panels show the merged image. Scale bar = 10 µm.
(3.44 MB PDF)

Figure S6 Mutant F12 and A36 proteins are stable in VACV infected cells. HeLa cells were infected at 5 pfu/cell with vΔF12L or vΔA36R for 2 h. Cells were then were transfected with plasmids expressing HA tagged WT or mutant F12 or A36 proteins (F12-WT-HA, F12-AAA-HA, A36-WT-HA, and A36-AAA-HA). 10 h later the cells were lysed and lysates were analysed by SDS-PAGE and immunblotting with anti-HA mAb. The positions of molecular size markers are indicated in kDa.
(0.65 MB PDF)

Figure S7 Conservation of the WD KBS in A36 chordopoxviruses orthologues. Alignment of the WD kinesin-1 binding site in A36 chordopoxviruses orthologues with the consensus sequence (L-N-W-D-N-+-+). Accession numbers are SWPV 123 (NP_570283), MYXV m125R (NP_051839), EMV (AJ315003), GTPV gp121 (YP_001293318), VACV WR A36 (YP_233041), YLDV 126R (NP_073511), DPV gp136 (YP_002302476).
(0.61 MB PDF)

Figure S8 The A36 WD motif is not required for recruitment of kinesin-1 to IEV or for virion transport to the cell surface. HeLa cells were infected at 5 pfu/cell with vΔA36R-EGFPA5L for 2 h. Cells were then transfected with plasmids expressing A36-WT-HA or A36-AAA-HA for 10 h. Cells were stained with mAbs against HA (blue) and KHC (red), and EGFP were visualized directly. Cells were viewed by confocal microscopy. The merged image is shown on the right. The insets show ×2.5 enlargements of the boxed regions and illustrate that both A36-WT-HA and A36-AAA-HA co-localise with KHC and virus particles. (B) Cells were treated as in (A) and were counterstained with phalloidin to label filamentous actin (red). Numerous actin tails are visible in cells transfected with A36-WT-HA and A36-AAA-HA. Scale bars = 10 µm.
(5.65 MB PDF)

Video S1 Tilt series electron microscopy showing vΔF12L arefully wrapped. Images were acquired from a 90-nm thin section stained with Reynold's lead citrate, 11 images at 10 degree increments were captured +/− 50 degrees tilt angle. Images were aligned with Adobe Photoshop CS4 and made into a movie using GIF Movie Gear and further compressed with Windows Media Encoder. Selected images are shown in Figure 10 (B,F,J,N), the complete animated tilt series shows areas of the virion that appear not to be wrapped with a double membrane are indeed fully associated with enveloping membrane when perpendicular to the electron beam.
(0.25 MB WMV)
