# Supplementary figures and images for: Vaccinia Protein F12 Has Structural Similarity to Kinesin Light Chain and Contains a Motor Binding Motif Required for Virion Export
Source: PLoS Pathog. 2010 Feb 26;6(2):e1000785. doi: 10.1371/journal.ppat.1000785 (PMC2829069; doi:10.1371/journal.ppat.1000785)

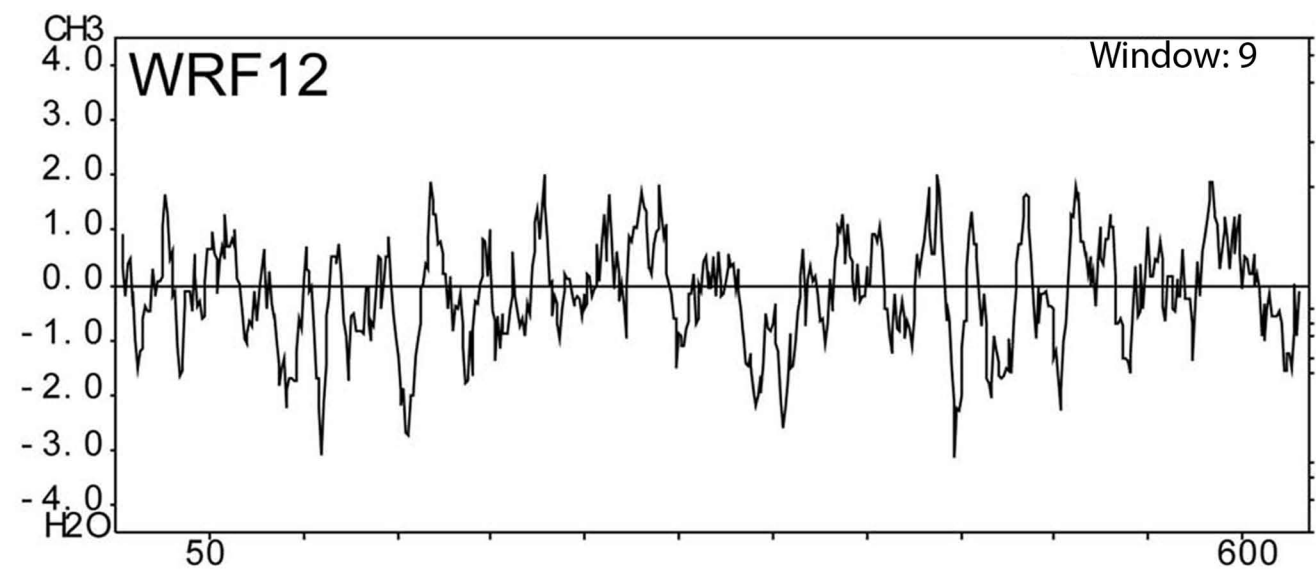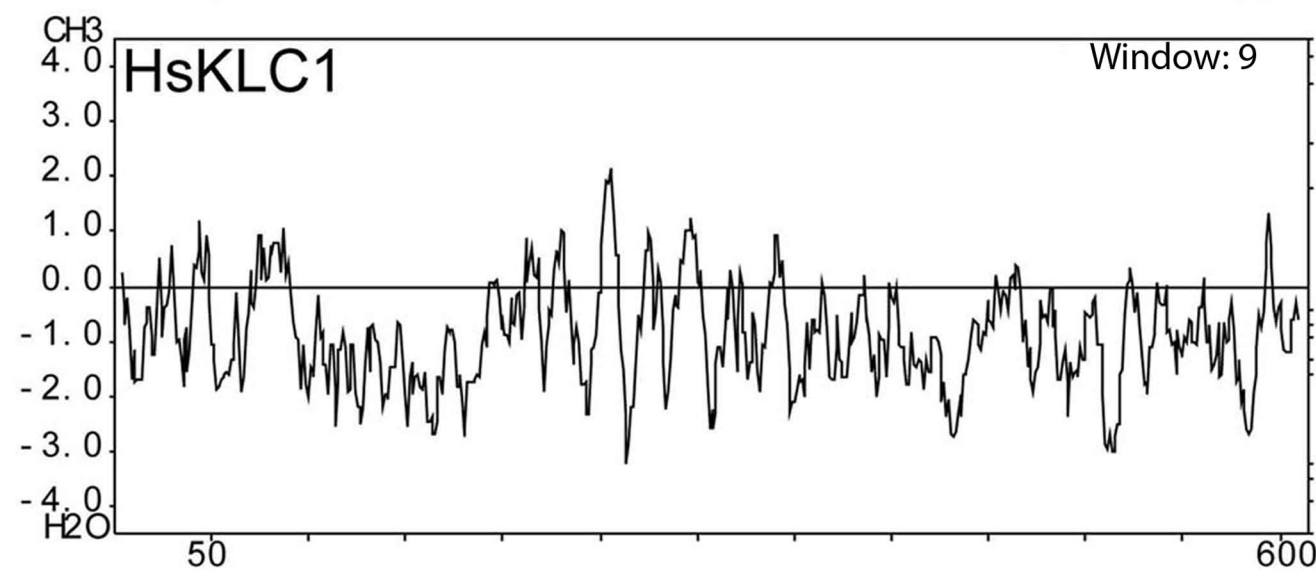

Supplement: Figure S1 — Kyte-Doolittle hydropathy plots of the VACV WR F12 and Homo sapiens KLC1 (HsKLC1). Note VACV WR F12 and HsKLC are similar in length and exhibit a similar oscillating hydrophobicity profile. (0.83 MB PDF) [file ppat.1000785.s001.pdf]

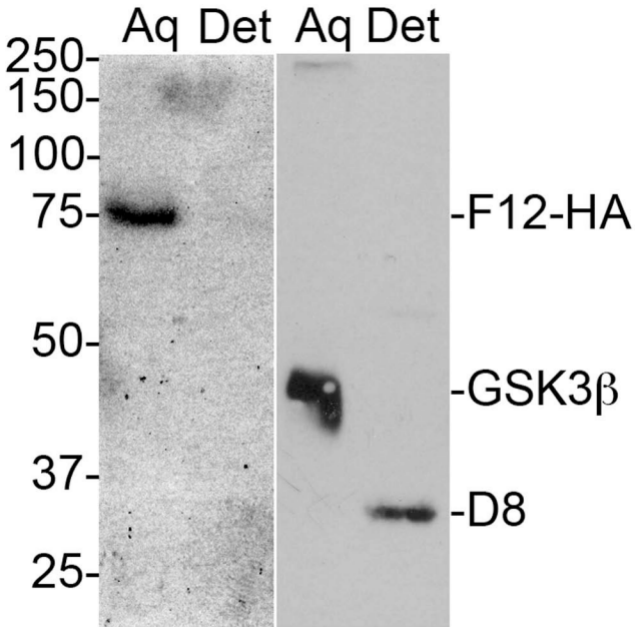

Supplement: Figure S2 — F12 is a cytosolic protein. F12-HA TritonX-114 partitioning. HeLa cells were infected at 5 pfu/cell with vF12L-HA for 12 h. Cells were then collected, lysed and subjected to TritonX-114 partitioning (Materials and Methods). Proteins in the soluble aqueous (Aq) or membrane detergent (Det) fractions were resolved by SDS-PAGE and immunoblotted with mAbs against HA (F12-HA) (left panel), or VACV protein D8 (membrane protein) and GSK3β (cytosolic protein) (right panel). The positions of molecular size markers are indicated in kDa. (0.47 MB PDF) [file ppat.1000785.s002.pdf]

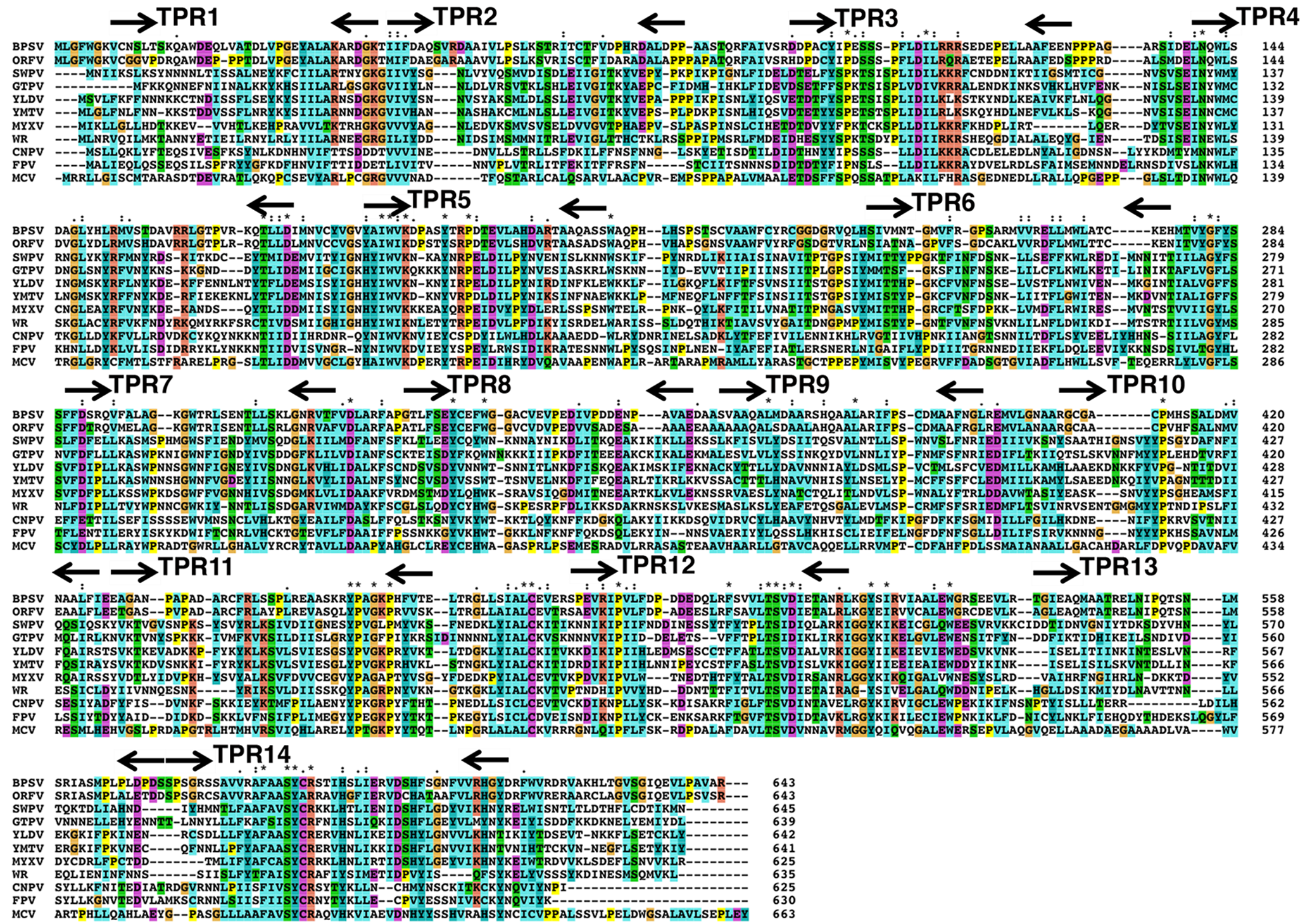

Supplement: Figure S3 — Alignment of F12 orthologues. F12 orthologues from different chordopoxvirus genera were aligned using Clustal-X and are displayed using the Clustal colour scheme (http://www.jalview.org/help/html/colourSchemes/clustal.html). The positions of TPRs numbered 1–14 are indicated above the alignment. The F12 TPRs that were aligned with KLC TPRs in Figure 1 are TPRs 6–10 and 12. Note the conservation of aromatic residues throughout the alignment and the lack of N-terminal heptad repeats, which mediate KHC binding by KLC. Abbreviations: BPSV, Bovine papular stomatitis virus; CNPV, canarypox virus; FWPV, fowlpox virus; GTPV, goatpox virus; MOCV, molluscum contagiosum; MYXV, myxoma virus; ORFV, orf virus; SWPV, swinepox virus; WR, VACV strain Western Reserve; YMTV, yaba monkey tumor virus. (4.95 MB TIF) [file ppat.1000785.s003.tif]

F12-HA

B5

Phalloidin

Merge

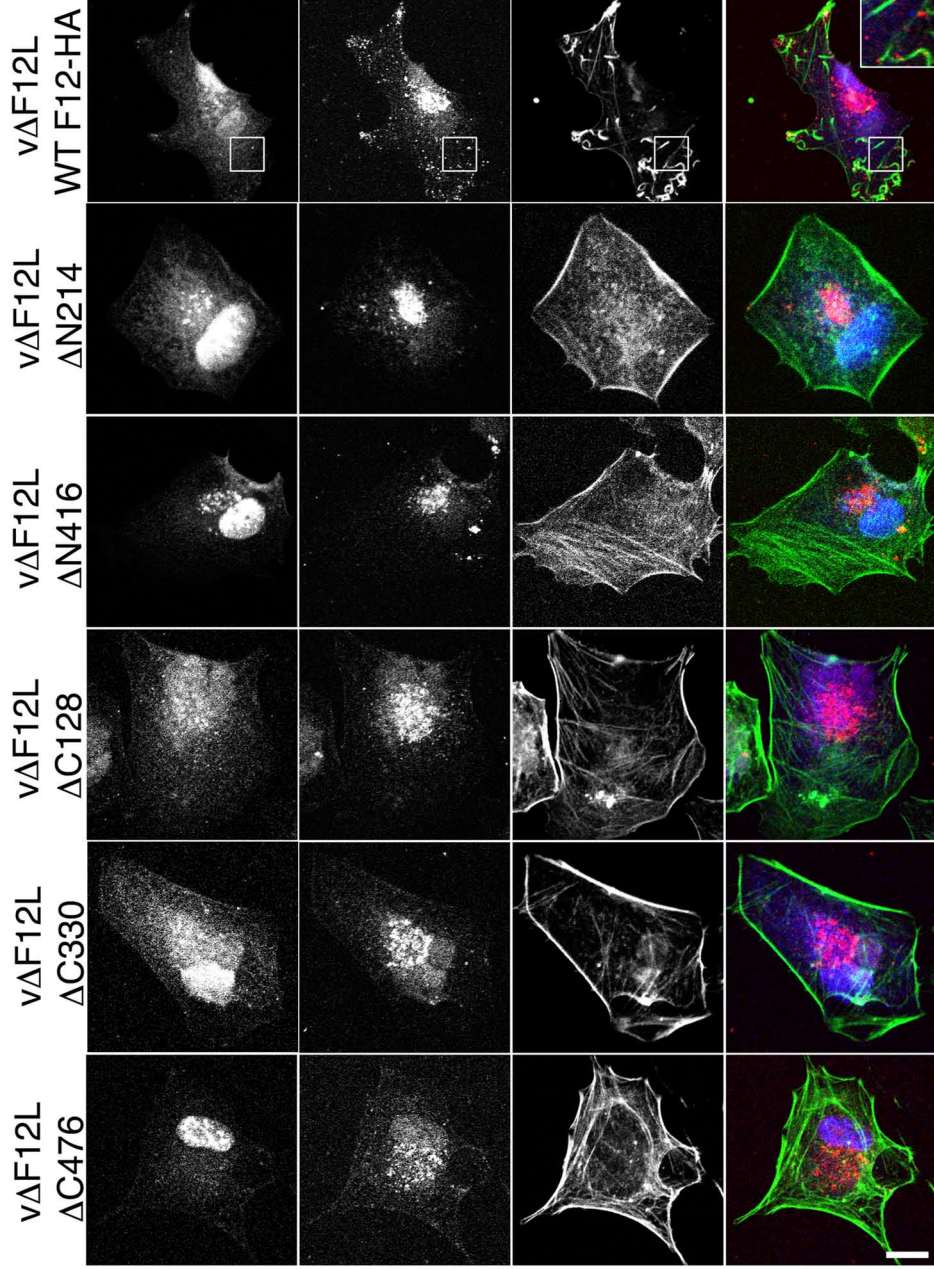

Supplement: Figure S4 — Full length F12 protein is required for IEV transport to cell periphery. HeLa cells were infected at 5 pfu/cell with vΔF12L and at 2 h p.i. were transfected with plasmids expressing full length F12L-HA or the indicated F12 mutants. The cells were fixed at 12 h p.i., permeabilised and stained with mAb against HA (blue), or B5 (red), and phalloidin-Alexa Fluor 488 to label filamentous actin (green). Cells were then viewed by confocal microscopy. The insert shows B5-positive virus particles at the tips of actin tails in cells transfected with the WT F12. Scale bar = 10 µm. (4.38 MB PDF) [file ppat.1000785.s004.pdf]

F12-HA

KHC

Merge

Mock

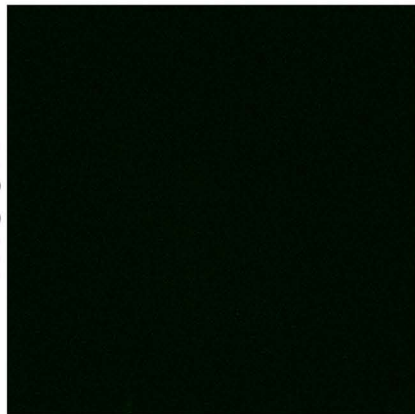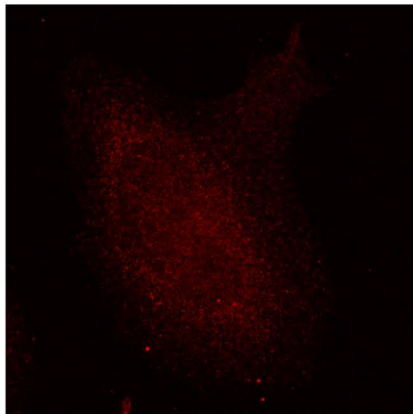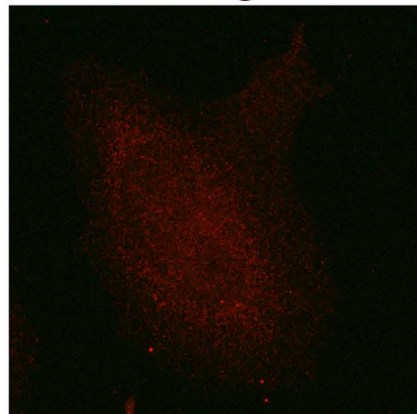

vF12L-HA

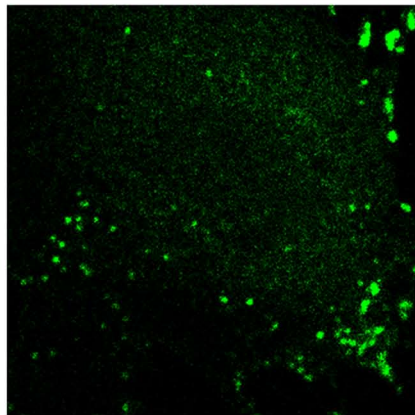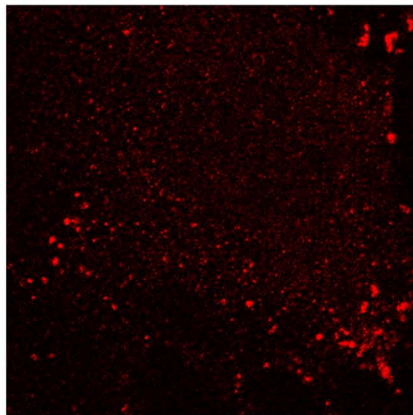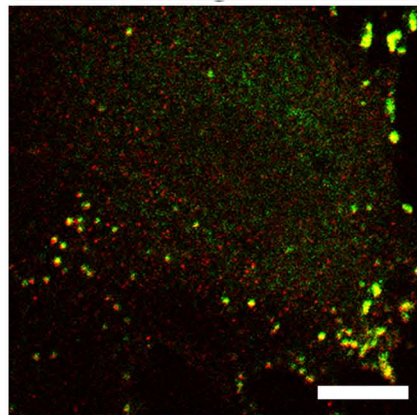

Supplement: Figure S5 — F12 co-localises with kinesin-1. Confocal microscopy showing colocalisation of F12-HA and KHC. HeLa cells were mock-infected or infected with vF12L-HA at 5 pfu/cell. At 12 h p.i. cells were permeabilised and stained with α-HA (F12-HA, green) and α-KHC (red). The right panels show the merged image. Scale bar = 10 µm. (3.44 MB PDF) [file ppat.1000785.s005.pdf]

F12-WT-HA

F12-AAA-HA

A36-WT-HA

A36-AAA-HA

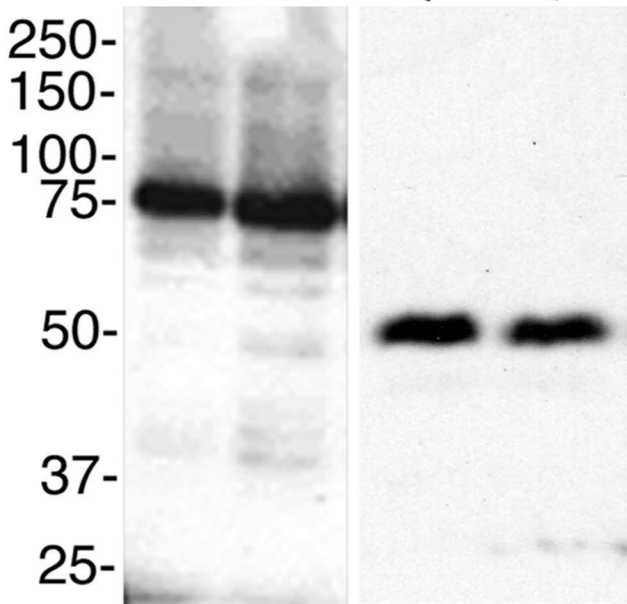

Supplement: Figure S6 — Mutant F12 and A36 proteins are stable in VACV infected cells. HeLa cells were infected at 5 pfu/cell with vΔF12L or vΔA36R for 2 h. Cells were then were transfected with plasmids expressing HA tagged WT or mutant F12 or A36 proteins (F12-WT-HA, F12-AAA-HA, A36-WT-HA, and A36-AAA-HA). 10 h later the cells were lysed and lysates were analysed by SDS-PAGE and immunblotting with anti-HA mAb. The positions of molecular size markers are indicated in kDa. (0.65 MB PDF) [file ppat.1000785.s006.pdf]

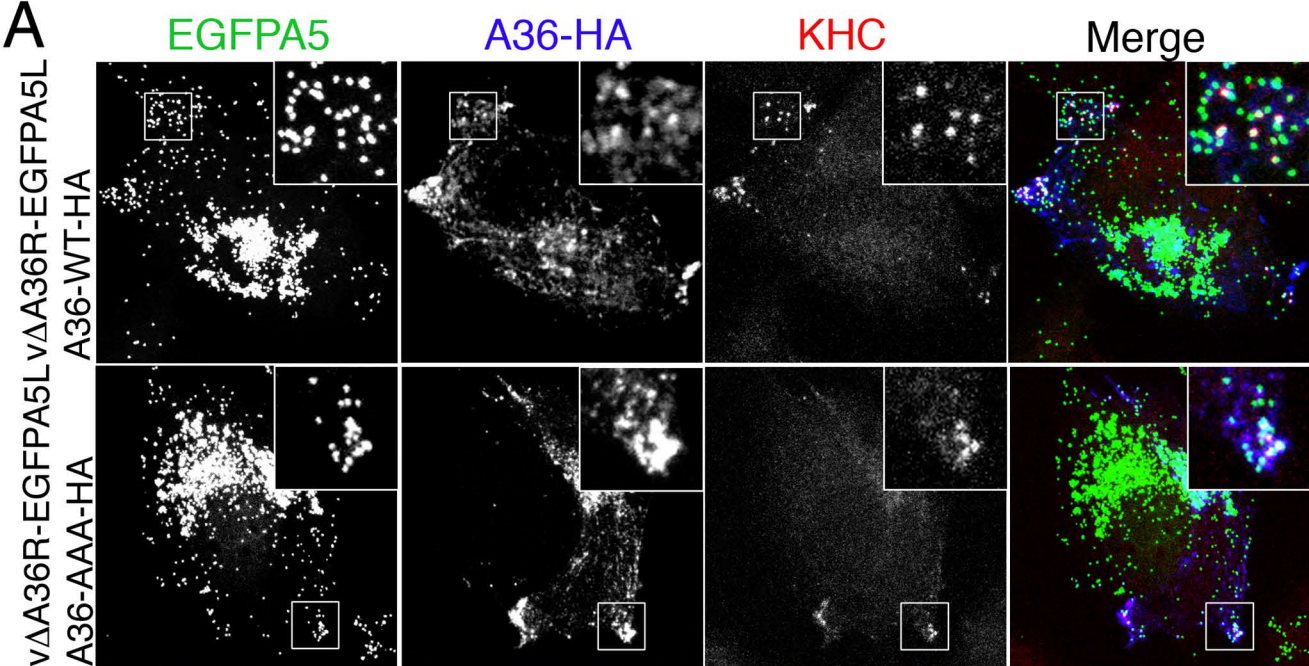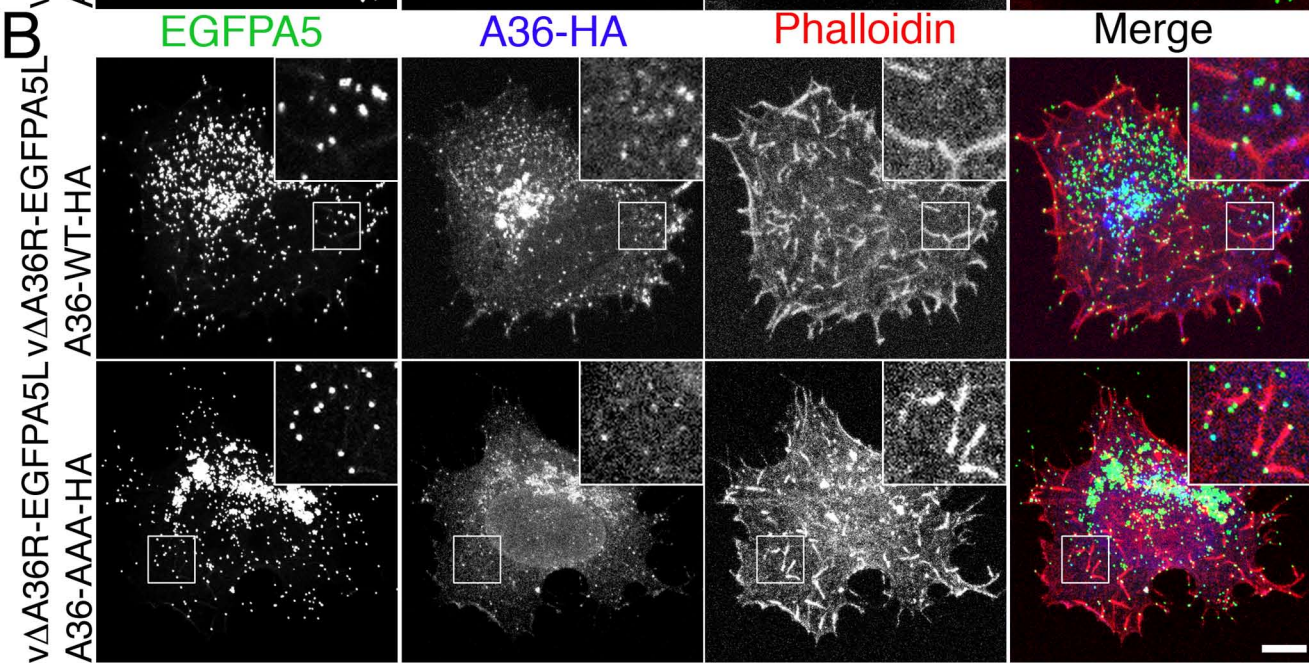

Supplement: Figure S8 — The A36 WD motif is not required for recruitment of kinesin-1 to IEV or for virion transport to the cell surface. HeLa cells were infected at 5 pfu/cell with vΔA36R-EGFPA5L for 2 h. Cells were then transfected with plasmids expressing A36-WT-HA or A36-AAA-HA for 10 h. Cells were stained with mAbs against HA (blue) and KHC (red), and EGFP were visualized directly. Cells were viewed by confocal microscopy. The merged image is shown on the right. The insets show ×2.5 enlargements of the boxed regions and illustrate that both A36-WT-HA and A36-AAA-HA co-localise with KHC and virus particles. (B) Cells were treated as in (A) and were counterstained with phalloidin to label filamentous actin (red). Numerous actin tails are visible in cells transfected with A36-WT-HA and A36-AAA-HA. Scale bars = 10 µm. (5.65 MB PDF) [file ppat.1000785.s008.pdf]
