# Supplementary material for: Vaccinia Protein F12 Has Structural Similarity to Kinesin Light Chain and Contains a Motor Binding Motif Required for Virion Export
Source: PLoS Pathog. 2010 Feb 26;6(2):e1000785. doi: 10.1371/journal.ppat.1000785 (PMC2829069; doi:10.1371/journal.ppat.1000785)
Supplement: Figure S7 — Conservation of the WD KBS in A36 chordopoxviruses orthologues. Alignment of the WD kinesin-1 binding site in A36 chordopoxviruses orthologues with the consensus sequence (L-N-W-D-N-+-+). Accession numbers are SWPV 123 (NP_570283), MYXV m125R (NP_051839), EMV (AJ315003), GTPV gp121 (YP_001293318), VACV WR A36 (YP_233041), YLDV 126R (NP_073511), DPV gp136 (YP_002302476). (0.61 MB PDF) [file ppat.1000785.s007.pdf]

SWPV	LNWDSGI
MYXV	PNWDPTI
WR	LIWDNES
YLDV	LNWDNEE
DPV	LNWDNDV
EMV	SDWDDHC
GTPV	LIWDDNN
